# Study protocol for an effectiveness-implementation hybrid trial to evaluate a health promotion intervention in parents and their 5-year-old child: Saga Stories in health talks in Swedish child healthcare

**DOI:** 10.1186/s12889-022-14549-z

**Published:** 2022-11-25

**Authors:** Christine Delisle Nyström, Maria Henström, Susanne Andermo, Gerd Almquist-Tangen, Kristin Thomas, Marie Löf

**Affiliations:** 1 Department of Biosciences and Nutrition, Karolinska Institutet, Neo, 141 83 Huddinge, Sweden; 2 Department of Neurobiology, Care Sciences and Society, Division of Nursing, Karolinska Institutet, 141 83 Huddinge, Sweden; 3 Department of Global Public Health, Karolinska Institutet, 171 77 Stockholm, Sweden; 4 Department of Physical Activity and Health, The Swedish School of Sport and Health Sciences, 114 33 Stockholm, Sweden; 5 Department of Pediatrics, Institute of Clinical Sciences, The Sahlgrenska Academy, University of Gothenburg, 405 30 Gothenburg, Sweden; 6 Child Health Care Unit, Region Halland, 301 80 Halmstad, Sweden; 7 Department of Health, Medicine and Caring Sciences, Division of Society and Health, Linköping University, 581 83 Linköping, Sweden

**Keywords:** Child healthcare, Health promotion, Lifestyle behaviours, Parental self-efficacy, Pre-school

## Abstract

**Background:**

Unhealthy lifestyle behaviours such as a poor diet, inadequate physical activity, and excessive screen time have been shown to be established in childhood and track into adulthood, demonstrating the need for health promotion interventions in the pre-school years. The overall aim of this project is to: (i) evaluate the effectiveness of `Saga Stories in health talks´ within child healthcare (CHC) on parental self-efficacy to promote healthy diet, physical activity, and screen time behaviours in their child; children’s intake of key dietary indicators and screen time and (ii) evaluate and explore the implementation of `Saga Stories in health talks´ with regards to acceptability, appropriateness, feasibility, fidelity, adoption, sustainability, satisfaction, and usage.

**Methods:**

A hybrid type I effectiveness-implementation trial will be conducted. A cluster randomized controlled trial will be used to assess the effectiveness of `Saga Stories in health talks´ in 42 CHC centers across six regions in Sweden. `Saga Stories in health talks´ consists of material for CHC nurses to use to facilitate the health talk with both the child and parent(s) and is complemented with take-home material. Parent and child dyads are recruited (*n* = 450) from participating CHC centers when they attend their 5-year routine visit. The intervention group receives the health talk using Saga Stories and take-home material, whereas the control group receives the standard health talk. The primary outcome is parental self-efficacy to promote healthy diet, physical activity, and screen time behaviours in their child and secondary outcomes include children’s intake of key dietary indicators and screen time. All outcomes are assessed at baseline and 2-months post-intervention. The implementation outcomes that will be assessed are: acceptability, appropriateness, feasibility, satisfaction, usage, fidelity, adoption, and sustainability (assessed quantitatively and qualitatively).

**Discussion:**

The Swedish National Board of Health and Welfare have identified the need of more material, education, and working methods for promoting healthy lifestyle behaviours in CHC. Following this trial `Saga Stories in health talks´ has great potential to be implemented in CHC across Sweden to aid nurses to promote and support healthy lifestyle behaviours in pre-school children and their families.

**Trial registration:**

ClinicalTrials.gov, NCT05237362. Registered 2 February 2022.

## Background

Unhealthy lifestyle behaviours (e.g., a poor diet, inadequate physical activity, and excessive screen time) have been shown to be established in childhood and track into adulthood [1, 2]. According to the 2021 Generation Pep report, including a representative sample of Swedish children and youth aged 4-17 years, only 10 and 20% of children and youth met the dietary recommendations for fruit and vegetables and fish intake as well as physical activity, respectively. Furthermore, 54% of parents of 4-6 year olds reported 1-2 hours of screen time the previous day and another 14% reported 3-4 hours of screen time [3]. Parental self-efficacy has been found to be associated with children’s dietary, physical activity, and screen time behaviours [4, 5], thus there is a need for health promotion interventions in the pre-school years to aid parents in encouraging healthy behaviours and minimizing unhealthy ones.

Child healthcare (CHC) in Sweden reaches 99% of all children [6] and CHC nurses meet parents and children continuously from birth until 5 years of age at regular check-ups [7]. Thus, this makes CHC an ideal arena for health promotion interventions aimed at promoting healthy lifestyle behaviours in families with young children. Furthermore, the Swedish National Board of Health and Welfare have identified the need for more material, education, and working methods for promoting healthy lifestyle behaviours within CHC [6]. This was further emphasized in a recent study where the majority of Swedish CHC nurses reported not feeling comfortable discussing screen time with parents. They stated that they felt that this is a new topic and do not have enough material to support their discussions [8].

Within CHC in Sweden, health talks and guidance on promoting healthy lifestyle behaviours are part of routine visits from 3 months of age [7]. However, previous research has indicated that lifestyle behaviours such as dietary habits, physical activity, sleep, and screen time are infrequently discussed at routine visits in CHC [9]. As health talks vary depending on the needs of the family, a variety of scientifically evaluated material needs to be available to CHC nurses in order for them to be able to comfortably and effectively have a dialogue with parents and their child regarding lifestyle behaviours.

## Aims

The aim of this protocol is to describe the study design and methodology of `Saga Stories in health talks´. The main objectives are:

(i) to evaluate the effectiveness of `Saga Stories in health talks´ on (a) parental self-efficacy to promote healthy diet, physical activity, and screen time behaviours in their child (primary outcome); (b) children’s intake of fruit and vegetables as well as sweet beverages (secondary outcomes); and (c) children’s screen time (secondary outcome).
(ii) to evaluate and explore the implementation of `Saga Stories in health talks´ with regards to acceptability, appropriateness, feasibility, fidelity, adoption, sustainability, satisfaction, and usage.

## Methods

**Fig. 1:**
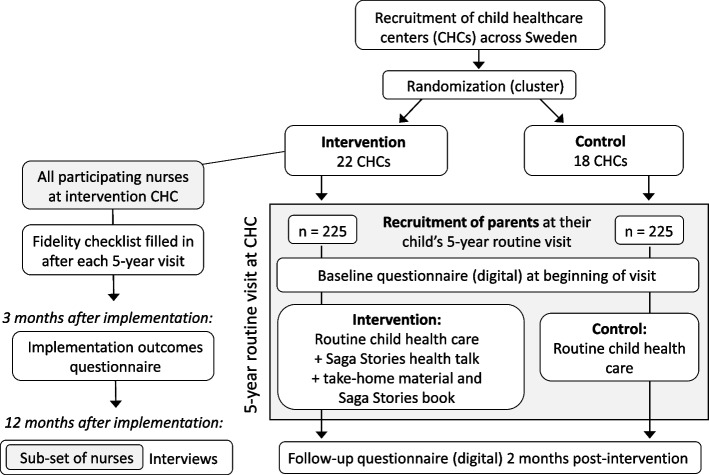
Flow chart of the `Saga Stories in health talks´ study design

This project will use a hybrid type I effectiveness-implementation trial design, as our primary focus is effectiveness and our secondary focus is implementation [10]. A mixed-methods approach will be used to collect data and the `Outcomes for Implementation Research´ [11] evaluation framework was used to inform the evaluation plan. The study design is shown in Fig. 1.

### Effectiveness evaluation

#### Study design, recruitment, and participants

`Saga Stories in health talks´ is a cluster randomized controlled trial which will be conducted in six regions throughout Sweden (Dalarna, Norrbotten, Värmland, Västernorrland, Västra Götaland, and Örebro). The intervention group receives the health talk using Saga Stories and take-home material, whereas the control group receives the standard health talk. Eligible families are provided with oral and written information regarding the study from their CHC nurse at the routine five-year check-up. If the accompanying parent consents to participate they will scan a QR code to sign the informed consent using BankID (a secure digital signature) and directly after they will fill in the baseline questionnaire. To be included in this study, the accompanying parent needs to be able to understand and read Swedish sufficiently well in order to provide informed consent and partake in the Saga Stories health talk. There are no exclusion criteria. This study is reported according to the SPIRIT 2013 statement [12] and Table 1 shows a trial participant timeline.

**Table 1:** Participant timeline according to SPIRIT

	STUDY PERIOD			
	Enrollment	Allocation	Post-allocation	Close-out
Time point	0	0	2 months	2 months
	5-year routine visit			
**Enrollment**				
Eligibility screen	X			
Informed consent	X			
Allocation (cluster)		X		
**Interventions**				
Intervention		X		
Control		X		
**Assessments**				
Baseline questionnaire	X			
Child height/weight	X			
Self-efficacy questionnaire	X		X	X
Intervention evaluation			X	X

#### Randomization and blinding

Forty-two CHC centers with 1-7 nurses per center were randomly assigned to the two treatment groups (22 CHC centers in the intervention and 20 in the control group). To ensure balance, we stratified the randomization by geographical regions and center size. Two CHC centers dropped-out before the trial was initiated due to high absenteeism because of the COVID-19 pandemic. Therefore, there is 22 CHC centers in the intervention group and 18 in the control group. Due to the nature of the trial, CHC nurses and participants cannot be blinded; however, outcome assessors are blinded to the treatment allocation of the participating families.

#### Intervention

**Fig. 2:**
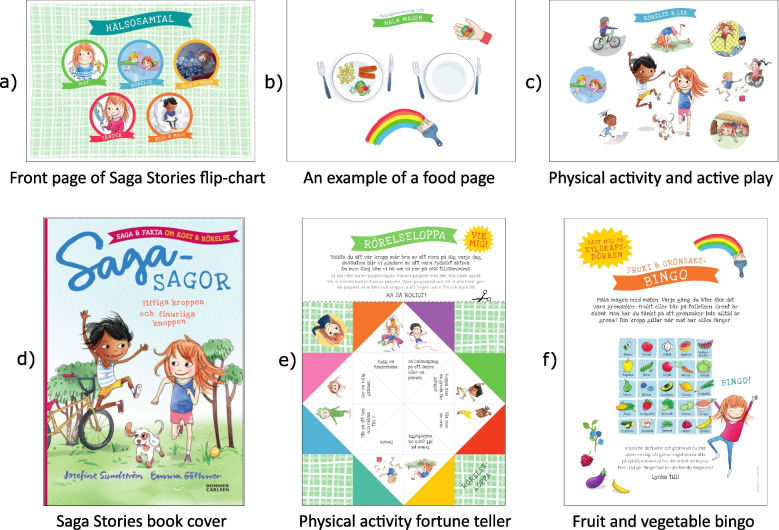
Screen shots of the Saga Stories material illustrated by Emma Göthner. The top row shows example pages of the Saga Stories flip chart used by the child healthcare nurses to facilitate the health talk at the five-year routine visit. These include a) the front page illustrating the four topics (food, physical activity and sedentary behaviour, sleep, dental health, and bathroom habits); b) an example from the healthy food habits pages, and c) an example of the physical activity and sedentary behaviour pages in the flip chart. The bottom row shows the take-home material that is given to the families at the visit: d) the Saga Stories book, e) the physical activity fortune teller, and f) the fruit and vegetable bingo

At the 5-year routine visit at primary (CHC), health talks where nurses provide information about healthy lifestyle behaviours (e.g., diet, physical activity, screen time etc.) are mandatory in all regions in Sweden. `Saga Stories in health talks´ consists of material for CHC nurses to use as a tool to facilitate the health talk with both the child and parent(s) and is complemented with take-home material for the families. Every nurse will receive a large flipchart with colourful pictures and text to facilitate their health talk at the five-year check-up. The flipchart includes information and suggested questions regarding food, physical activity and sedentary behaviour, sleep, dental health, and bathroom habits. Depending on the needs of the family the nurse can focus on the subjects most applicable to that family and tailor the health talk using the ‘Saga Stories’ material. For instance, if a family feels they are struggling with food and do not perceive physical activity and sedentary behaviour as a problem the majority of the health talk may be focused upon food. Thus, some health talks will include all subjects while others may only focus on one or two subjects. At the conclusion of the check-up the children receive the book `Saga Stories: Your amazing body and brain´, which is an inspirational and educational book which the parents are encouraged to read together with their child. The parents are given a hand-out showing an ideal 24-hour period with regards to time spent sleeping, playing, being physically active, and using screens for five-year olds. They also receive games for the child to take home (fruit and vegetable bingo and a physical activity fortune teller), which are intended to promote healthy lifestyle behaviours. The intention of the games and book is to create curiosity and interest in the child (together with their parents) for new foods and active play. Figure 2 shows pictures of the intervention material and Table 2 provides a more detailed description of the intervention material.

**Table 2:** Detailed description of the Saga Stories in health talks material

Material included in Saga Stories in health talks	Purpose of the material	What the material consists of	Target behaviour in relation to the study
Flipchart for child healthcare nurse	A flipchart to help the nurse facilitate the health talk at the 5-year routine visit at primary child healthcare.	The flipchart consists of colourful pictures and text covering the following topics: food; physical activity and sedentary behaviour; sleep; dental health; and bathroom habits.	- Parental self-efficacy to promote healthy diet, physical activity, and screen time behaviours - Children’s intake of fruit, vegetables, and sweet drinks - Children’s screen time
Saga Stories: Your amazing body and brain	An inspirational and educational book covering the topics covered in the health talk that parents are encouraged to read together with their child at home.	A book featuring a story about the characters Saga and Samir building an obstacle course together. The book includes easy facts about the body and what happens when we move, but also includes a part with information directed only to parents.	- Parental self-efficacy to promote healthy diet, physical activity, and screen time behaviours - Children’s intake of fruit, vegetables, and sweet drinks - Children’s screen time
The “Pep” 24-hour day poster	To give the parents an overview of what a typical 24-hour period should look like for a 5-year-old child with regards to sleep and rest, play and development, movement and physical activity, and screen time.	Hand-out (A4 size)	- Parental self-efficacy to promote healthy diet, physical activity, and screen time behaviours - Children’s screen time
Fruit and vegetable bingo	To promote healthy dietary behaviours through a game by encouraging children to try a wide variety of fruits and vegetables without pressure. This material is targeted to the parents and the child.	Hand-out (A4 size)	- Parental self-efficacy to promote healthy diet, physical activity, and screen time behaviours - Children’s intake of fruit, vegetables, and sweet drinks
Physical activity fortune teller	To promote physical activity and reduce sedentary time through play. This material is targeted to the parents and the child.	Hand-out (the child and the parent fold it into a fortune teller)	- Parental self-efficacy to promote healthy diet, physical activity, and screen time behaviours

`Saga Stories in health talks´ was created by Generation Pep (a non-profit organization working with health promotion in Swedish children and youth [13]) together with the publisher Bonnier Carlsen, and author Josefin Sundström. This material was also developed in collaboration with CHC nurses, dieticians, pediatricians, and researchers in an iterative process. In 2021, the `Saga Stories in health talks´ material was pilot tested in 11 CHC centers in three regions in Sweden (Halland, Sörmland, and Östergötland). Semi-structured interviews were conducted with a sample of participating CHC nurses (*n* = 17) and parents (*n* = 11) who received the health talk using the Saga Stories material. The information that was obtained from the qualitative pilot studies was used to adapt the Saga Stories material and inform the study design. The results from the pilot work will be reported elsewhere.

#### Outcome measures

##### Primary outcome

Parental self-efficacy regarding the parent’s own ability to promote healthy lifestyle behaviours in their five-year old child is the primary outcome. This is assessed using an electronic version of the validated `Parental self-efficacy for promoting healthy dietary and physical activity behaviours in their children´ questionnaire. This questionnaire includes 16 questions divided into three factors (i.e., self-efficacy for promoting healthy dietary behaviours in children, self-efficacy for limit-setting of unhealthy dietary or physical activity behaviours, and self-efficacy for promoting healthy physical activity behaviours) [14]. This questionnaire will be filled in online by the same parent (i.e., the parent that accompanied the child to the five-year check-up) at baseline and post-intervention (i.e., two months after the five-year check-up).

##### Secondary outcomes

A modified version of the Swedish National Board of Health and Welfare’s survey regarding health behaviours [15] is used to assess dietary outcomes. The amount of fruits and vegetables are assessed as the average number of standardized portions per day over the past month, and the intake of sweet beverages as the average frequency per day. The amount of time spent watching screens (e.g., phones, tablets, TV, and computer) is assessed in minutes per day on a typical day that the child is at home and not at pre-school.

At baseline, the accompanying parent will also complete a brief electronic demographic questionnaire which includes questions regarding their age, sex, and socioeconomic position as well as their child’s date of birth, sex, country of birth, weight, and height (measured at the five-year check-up).

##### Sample size

We calculated the sample size required to achieve a statistical power of 80% to detect a four-point difference in parental self-efficacy between the two treatment groups. The power was for a Wald test of the coefficient of the group variable in linear random intercept model where the 6-month change in outcome is the dependent variable and the group indicator is the only covariate. The linear random-intercept model included a center-specific normal random intercept, which accounted for the within-center stochastic dependence of the data. The estimated sample size was obtained by generating 1000 pseudo-random Monte-Carlo samples under an assumed intra-center correlation of 0.03 and the two specified group differences. The assumed fixed regression coefficients and the regression residual variance were estimated using unpublished parental self-efficacy data from the MINISTOP trial [16]. A total of 368 parent child dyads (*n* = 184 per arm) are needed. Assuming a 20% drop-out rate [16, 17], a minimum of 450 children will be recruited, i.e., 225 per group.

##### Statistical analyses

All analyses will be conducted according to the intention-to-treat method. The primary analysis will be completers only, as long as the missing data is considered missing completely at random. Missing data will be imputed using multiple imputations with chained equations [18]. To contrast differences in parental self-efficacy, dietary intake, and screen time between treatments, we will use linear random-effects models, specifying the CHC center as a random effect to account for clustering. The models will be estimated unadjusted and adjusted for child age (continuous) and sex (boy vs. girl). Interaction analyses will also be conducted to investigate if the interventional effect differs with regards to the participating parents’ age, education status, country of birth, and body mass index (BMI).

### Implementation evaluation

#### Study design

The implementation evaluation is taking place in the 22 CHC centers allocated to the intervention arm. All participating nurses were informed (orally and in text) about the evaluation and provided informed consent before participating. Prior to the implementation of `Saga Stories in health talks´, all participating CHC nurses attended a half-day online education on how to use the Saga Stories material in their health talks, as well as how to recruit parents to the study. During the entire intervention period the nurses have access to an online folder with study information, commonly asked questions, as well as the `Saga Stories in health talks´ materials for printing when needed. The nurses also have the possibility to contact the research team by phone or email if they have other questions pertaining to the study or intervention material. Once a month, the nurses receive a newsletter with information on the status of recruitment and reminders on how to use the Saga Stories materials. In addition, two or three times per year, all nurses in the intervention group are invited to participate in an online discussion forum for 1 h, to share experiences with each other. These forums are voluntary and focus on different topics such as talking about challenging or sensitive topics e.g., children’s growth chart and BMI.

#### Outcomes

##### Acceptability, appropriateness, and feasibility

Three months following the implementation of `Saga Stories in health talks´ CHC nurses in the intervention group will complete the questionnaire developed by Weiner et al. [19] to assess the acceptability, appropriateness, and feasibility of `Saga Stories in health talks´. The questionnaire consists of four questions for each outcome measured on a five-point Likert scale with the following answer options: Completely disagree, Disagree, Neither agree nor disagree, Agree, or Completely agree. Response scores will be created by setting a value of 1 to 5 for each answer alternative and summing the score to create a sub-score for each outcome. Thus, a higher score will indicate greater acceptability, appropriateness, or feasibility. The questionnaire has been shown to have good validity and reliability [19].

##### Fidelity and adoption

A checklist will be used to assess implementation fidelity. Following every five-year check-up, each CHC nurse will use the checklist to indicate whether they have followed the implementation procedures. Within the checklist there is room to provide open ended notes. The checklist includes the following questions: 1. Did you use the ‘Saga Stories’ material (Yes or No); 2. If you did not use the material, why not? (Lack of time, Used other material instead, The family did not want the health talk, Prioritised something else during the visit, Other - please specify); 3. Which parts of the flipchart did you use during the health talk (Food, Physical activity and sedentary behaviour, Sleep, Dental health, and Bathroom habits); 4. Did you send the take home material with the family? (Saga Stories book, Fruit and vegetable bingo, Physical activity fortune teller, Ideal 24-hour period, None of them). Adoption is assessed through the CHC nurses’ documentation within the checklist. Here they will be asked to record when they have had a five-year check-up and whether or not they have used the Saga Stories material to facilitate the health talk. Fidelity and adoption will be assessed throughout the implementation period.

##### Sustainability

One year after implementation, semi-structured interviews will be held with a purposeful sample of CHC nurses to investigate key factors influencing sustained implementation. The results of the interviews will be used to investigate if future adjustments are needed for the ‘Saga Stories in health talks’ material as well if there is a need to refine the implementation procedures before Saga Stories is implemented on a larger scale.

##### Satisfaction & usage

Parental satisfaction and usage of `Saga Stories in health talks´ will be assessed using a digital questionnaire 2 months after they have received the Saga Stories health talk at CHC. The questionnaire includes both multiple choice and open-ended questions. Examples of some of the questions are: 1. Do you remember talking about healthy habits at your child’s 5-year routine visit? (Yes, Maybe – do not remember, No); 2. Which parts of the health talk did you talk about (Food, Physical activity and sedentary behaviour, Sleep, Dental health, and Bathroom habits); 3. How much did your child participate in the health talk? (My child did not want to participate, My child participated a little, My child kind of participated, and My child fully participated and was engaged); and 4. How was your experience with the health talk with the Saga Stories material? (Very good, Good, Ok, Not so good, Very bad).

##### Data analysis

Descriptive statistics will be used for the questionnaires and checklists. Semi-structured interviews with CHC nurses will be recorded (audio) and transcribed verbatim following the process described by Ersson et al. [8]. Data will be analyzed using inductive thematic analysis [20, 21].

### Ethics approval

This study was approved by the Swedish Ethical Review Authority (Dnr 2021-06106-01). Written informed consent will be collected from participating parents, and CHC nurses in the intervention group (i.e., those participating in the implementation evaluation).

### Trial status

Recruitment for this trial was initiated in February 2022 and is progressing according to the time plan.

## Discussion

To date, there is a lack of material for promoting healthy lifestyle behaviours for CHC nurses that have been evaluated with regards to both effectiveness and implementation. Thus, the `Saga Stories in health talks´ trial will investigate whether a working tool plus take-home material at the five-year routine visit can be used to improve parental self-efficacy for promoting healthy lifestyle behaviours as well as children’s diet and screen time behaviours. Furthermore, we aim to evaluate the implementation of `Saga Stories in health talks´ in terms of acceptability, appropriateness, feasibility, fidelity, adoption, sustainability, satisfaction, and usage.

In Sweden, there is a variety of material available for CHC nurses to support the health talks with families at the routine visits at CHC [22]; however, very few have been scientifically evaluated. `The child-centered health dialogue for the prevention of obesity´ has recently been evaluated, where they investigated whether a structured dialogue at the four-year routine visit between the CHC nurse, the child, and the caregiver(s) improved BMI z-scores. The intervention for all four-year-old’s regardless of weight status comprised of using eight animated illustrations on healthy choices plus a discussion around the child’s growth. At the follow-up 1 year later no significant intervention effect was found on children’s BMI z-scores [23]. Furthermore, in non-participant observations during the four-year visit as well as in interviews with the participating children, the authors found that the children seemed to enjoy being actively involved in the conversation, but were not able to interpret the health messages as intended [24]. Therefore, in the current trial we have placed our health talk at the five-year visit in order for the children to hopefully have a better ability to discuss and comprehend the messages that are being discussed.

One of the main strengths of this trial is the type I hybrid-design which allows us to evaluate both effectiveness and implementation outcomes. Collecting both effectiveness and implementation outcomes simultaneously has the potential to expedite the translation of research results into routine care [10]. Additional strengths of this study include the randomized controlled trial design (effectiveness evaluation) and large sample size accounting for clustering on the CHC level. Furthermore, public health interventions are conventionally developed using a top-down approach and do not include the end-users in the development process [25]. A recent systematic review and meta-analysis on international healthcare literature found that co-creation is thought to improve health related outcomes such as health-promoting behaviours [26]. In this case CHC nurses requested material that they could use to facilitate their health talks with families. Thereafter, relevant stakeholders were brought on board to create the material together with the nurses. Thus, even though Saga Stories for use in health talks (i.e., a working tool plus take-home material) was not created using a specific theoretical background it was developed utilizing co-creation through involving end-users (CHC nurses and parents), stakeholders (Generation Pep, dieticians, and pediatricians), and academic researchers in an iterative process. A limitation of this study is that only parent and child dyads that can speak and read Swedish sufficiently well will be included in this study, which will limit the generalizability of the results. In Sweden, approximately 25% of children have a migrant background [27] and at visits within CHC anyone that does not speak Swedish has the right to an interpreter. A study by Ersson et al. [8] with Swedish CHC nurses has previously found that communication through an interpreter may affect how messages are delivered. As the Saga Stories in health talks intervention’s focus is the health dialogue between the nurse, parent(s), and child we have only included Swedish speaking participants to ensure the intervention is delivered as intended. This study is further limited through the use of self-reported outcomes for the effectiveness evaluation. The baseline questionnaire, including questions on both parental self-efficacy for promoting healthy lifestyle behaviours as well as the child’s dietary intake and screen time, will be evaluated at the beginning of the routine five-year check-up at baseline. This, coupled with the lack of blinding of nurses and parents could lead to social desirability bias. The research team carefully discussed theses limitations during the study design process; however, there was no way to avoid these and thus they should be taken into consideration when interpreting the results. It is also important to highlight that researchers with expertise in both clinical and implementation research have designed this trial together in order to optimize the hybrid design. This is important, as using hybrid designs can be challenging due to the use of different constructs, theories, and concepts between clinical and implementation researchers [10].

## Conclusions

Following this trial, `Saga Stories in health talks´ has great potential to be nationally available for all nurses to use in health talks with parents. Furthermore, after this trial there is the possibility to translate and culturally adapt the Saga Stories material into the most commonly spoken languages in Sweden (besides Swedish), i.e., English, Somali, and Arabic to further facilitate health talks with non-Swedish speaking families. Overall, if Saga Stories in health talks is found to be effective, appropriate, as well as feasible to implement, it can aid CHC nurses to promote and support healthy lifestyle behaviours in pre-school aged children and their families.

## Data Availability

Not applicable as this article does not present data.

## Abbreviations

CHC: Child healthcare
BMI: Body mass index
